# Urban agriculture matters for sustainable development

**DOI:** 10.1016/j.crsus.2024.100217

**Published:** 2024-09-27

**Authors:** Prajal Pradhan, Daya Raj Subedi, Kshitij Dahal, Yuanchao Hu, Prakriti Gurung, Sijal Pokharel, Sagar Kafle, Biplav Khatri, Sudeeksha Basyal, Monika Gurung, Aruna Joshi

**Affiliations:** 1 Integrated Research on Energy, Environment & Society (IREES), Energy and Sustainability Research Institute Groningen (ESRIG), University of Groningen, Groningen, the Netherlands; 2 Potsdam Institute for Climate Impact Research (PIK), Member of the Leibniz Association, P.O. Box 60 12 03, 14412 Potsdam, Germany; 3 MU Institute of Cooperation and Development (MICD), Mid-West University (MU), Nayabato, Lalitpur, Nepal; 4 School of Forestry, Beijing Forestry University, No. 35 Qinghua East Road, Haidian District, Beijing, China; 5 School of Sustainable Engineering and the Built Environment, Arizona State University, Tempe, AZ, USA; 6 School of Resources & Environmental Sciences, Wuhan University, 129 Luoyu Road, Wuhan 430079, China; 7 United Nations Educational Scientific and Cultural Organization (UNESCO), Kathmandu, Nepal; 8 Department of Biosystems Engineering, Auburn University, Auburn, AL 36849, USA; 9 Department of Agricultural Engineering, Purwanchal Campus, Institute of Engineering, Tribhuvan University, Gangalal Marg, Dharan 56700, Koshi Province, Nepal; 10 Ministry of Urban Development, Government of Nepal, Kathmandu, Nepal; 11 Natural Hazards Section, Himalayan Risk Research Institute, Bhaktapur, Nepal

**Keywords:** Sustainable Development Goals, SDGs, urban agriculture, synergies and trade-offs, SDG interactions, city farming, urban gardens, qualitative analysis, leveraging opportunities, hurdles

## Abstract

Urban agriculture can contribute to sustainable development. However, a holistic investigation is lacking to comprehend its positive and negative impacts on the Sustainable Development Goals (SDGs). Our systematic analysis of around 1,450 relevant publications on urban agriculture, screened from 76,000 records, fills this gap. We map and analyze the text in the literature for each SDG target and its associated positive or negative sentiments. Here, we report our results highlighting that urban agriculture is linked to all SDGs, with 142 and 136 targets having positive and negative sentiments. The mapped positive sentiments are around double the negative ones. We identify six leveraging opportunities urban agriculture provides for sustainable transformation with four hurdles to be resolved. Urban agriculture does not inherently contribute to sustainability. Its impacts rely on the adoption of specific practices. Realizing urban agriculture’s social, economic, and environmental functions to accelerate SDG progress requires tackling the hurdles.

## Highlights

• Urban agriculture does not inherently contribute to sustainability
• Urban agriculture can positively impact many SDGs but negatively affect some
• Six opportunities and four hurdles are behind SDG and urban agriculture linkages
• Resolving the hurdles enhances urban agriculture’s contributions to sustainability

## Science for society

Urban agriculture, i.e., farming activities within and around cities, provides many social, economic, and environmental benefits. However, it can also have negative impacts. We show that urban agriculture is linked to all Sustainable Development Goals with more positive impacts than negative ones. Six opportunities and four hurdles are behind these impacts. Urban agriculture can be more sustainable by leveraging these opportunities and tackling the hurdles.

## Graphical abstract

**Figure:**
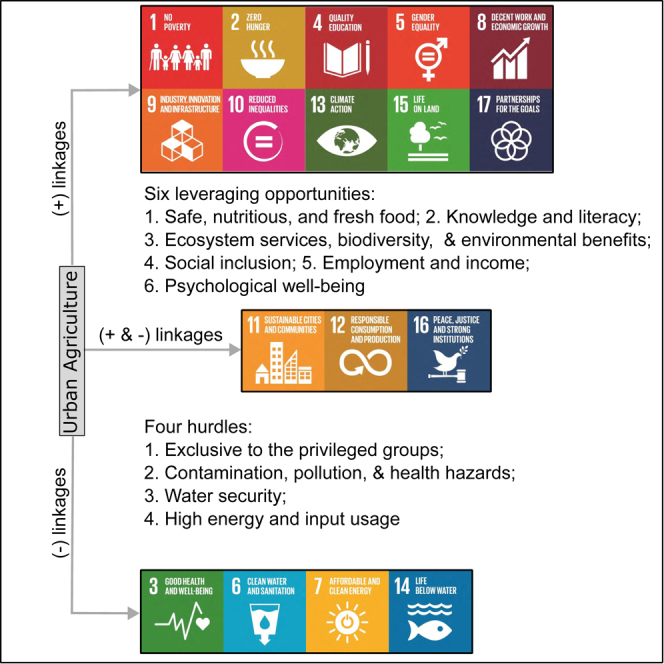

## Teaser

This study holistically investigates the positive and negative impacts of urban agriculture on the Sustainable Development Goals (SDGs). Its findings indicate that urban agriculture is linked to all SDGs, with 142 and 136 targets having positive and negative sentiments. Six opportunities and four hurdles are behind these linkages. Urban agriculture does not inherently contribute to sustainability. Its impacts rely on the adoption of specific practices. Realizing urban agriculture’s social, economic, and environmental functions to accelerate SDG progress requires leveraging the opportunities and tackling the hurdles.

## Introduction

Various farming activities prevail globally within cities and their surroundings, collectively termed urban agriculture. These activities encompass crop cultivation, horticulture, agroforestry, beekeeping, livestock rearing, and aquaculture.^1^ They are ground-based or building-integrated with or without space conditioning.^2^^,^^3^ These activities contribute to urban food security by producing 5%–10% of global food^1^ and employing 25%–30% of people living in urban areas.^4^ Besides food, urban agriculture has multiple economic, environmental, and social functions.^5^^,^^6^^,^^7^^,^^8^^,^^9^ These functions include livelihoods, health benefits, social space, green infrastructure, biodiversity, and ecosystem services.^9^ However, consuming urban-produced food could pose health risks due to soil and water contamination.^10^ Moreover, urban agriculture per se might not have multiple functions contributing to sustainability. So far, limited studies have investigated the opportunities and hurdles of urban agriculture for sustainable development.

Countries adopted the 2030 Agenda for Sustainable Development in 2015, consisting of 17 goals and 169 targets to transform our world into a sustainable and resilient path.^11^ So far, no country is on track to meet all the Sustainable Development Goals (SDGs).^12^ Achieving the 2030 Agenda would positively impact the lives and livelihoods of billions of people and tackle the existing socioeconomic and environmental crises. Doing so requires accelerating global sustainable development efforts based on understanding the roles of various sectors to leverage synergies and tackle trade-offs. For example, climate actions, food system transformation, energy transition, and urban transformation are crucial for the rapid progress of the SDGs.^13^^,^^14^^,^^15^ Urban agriculture, with multifunctionality, has the potential to impact sustainable development positively. However, this potential must be explored and accounted for comprehensively.^16^

Understanding urban agriculture’s positive and negative impacts on and linkages to the SDGs would help identify existing leveraging opportunities and hurdles to be resolved for accelerating sustainable development efforts. Studies apply quantitative, qualitative, or knowledge co-creation approaches to investigate these impacts and linkages.^17^ Besides understanding them, qualitative methods and knowledge co-creation enable deciphering mechanisms behind the impacts and linkages.^18^ Thus, promoting urban agriculture to contribute to the SDGs requires an evidence synthesis of its multiple functions linking to sustainable development. So far, urban agriculture reviews have focused on limited aspects, e.g., income, food security, crop productivity, and environmental benefits.^2^^,^^6^^,^^19^^,^^20^^,^^21^^,^^22^ A holistic and robust evidence synthesis encompassing urban agriculture’s role in accelerating sustainable development is lacking.

We fill the above-highlighted research gaps by conducting a literature analysis to understand the impacts of urban agriculture on the SDGs (see experimental procedures for details). Our analysis uses Pradhan and colleagues’ systematically searched and screened literature on urban agriculture.^9^ They screened 76,000 initial records on urban agriculture using a machine learning-supported approach, which resulted in 1,455 relevant full texts. We apply a text analysis approach supplemented with in-depth manual analysis to systematically map the text in the urban agriculture literature to each SDG target. For this, we adopt the SDG search queries from the Aurora Universities Network^23^ and convert them to Corpus Query Language. We extract SDG-related text in the urban agriculture literature by applying these queries using the “corpustools” package in the R programming language.^24^ Next, we conduct a sentiment analysis to determine if the extracted text highlights urban agriculture’s positive or negative associations with SDGs using R’s “tidytext” package. Lastly, we conduct an in-depth manual analysis of the extracted text and its sentiments, assessing and referring to the relevant document. In doing so, we compile the reasons behind urban agriculture’s positive or negative impacts on SDGs and related leveraging opportunities and hurdles.

Our study highlights that urban agriculture does not inherently contribute to sustainability, although it positively impacts many SDGs. The urban agriculture literature refers to more SDG targets with positive than negative sentiments. Leveraging opportunities and tackling hurdles associated with urban agriculture would enhance its contribution to sustainability.

## Results

### Urban agriculture and SDGs

**Figure 1:**
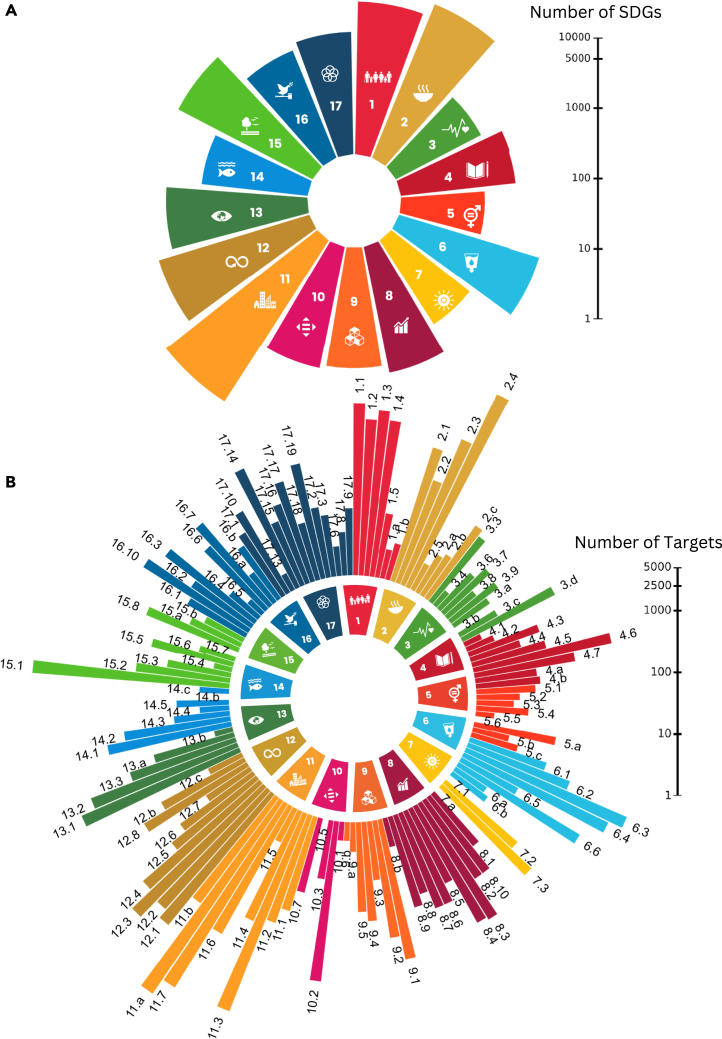
Frequency of SDGs and targets mapped in the urban agriculture literature We mapped the connection between SDGs and urban agriculture with a systematic analysis of around 1,450 relevant full texts screened from 76,000 initial records. The bar height represents the frequency in the log scale, i.e., the number of times an SDG or target is mapped in the relevant full text. The targets, e.g., 1.1, 1.2, and 1.3, are mentioned in the outer ring of the bars in (B). Table S3 provides the full names of SDGs and targets.

We map 17 SDGs and 143 out of 169 targets in the urban agriculture literature (Figure 1). This mapping reflects a broader connection between sustainable development and urban agriculture. However, their appearances in the literature vary, ranging from 60 to 12,000 times for the goals and from one to around 6,000 times for the targets. SDGs 2 (*zero hunger*), 11 (*sustainable cities and communities*), and 12 (*responsible consumption and production*) are the top three goals mapped in the urban agriculture literature, appearing at least 2,000 times. These appearances highlight urban agriculture’s potential associations in making cities more sustainable, ensuring food and nutrient security, and promoting alternative consumption and production. However, the top 10 mapped targets, appearing at least 500 times, also belong to other goals, namely, SDGs 1 (*no poverty*), 6 (*clean water and sanitation*), 13 (*climate action*), and 15 (*life on land*). They include targets 1.1 (*eradicate extreme poverty*), 2.4 (*sustainable food production and resilient agricultural practices*), 6.3 (*improve water quality, wastewater treatment, and safe use*), 11.7 (*provide access to safe and inclusive green and public spaces*), 13.1 (*strengthen resilience and adaptive capacity to climate-related disasters*), and 15.1 (*conserve and restore terrestrial and freshwater ecosystems*). These findings show broader linkages between urban agriculture across various SDGs and their targets besides the goals related to cities and food systems.

**Figure 2:**
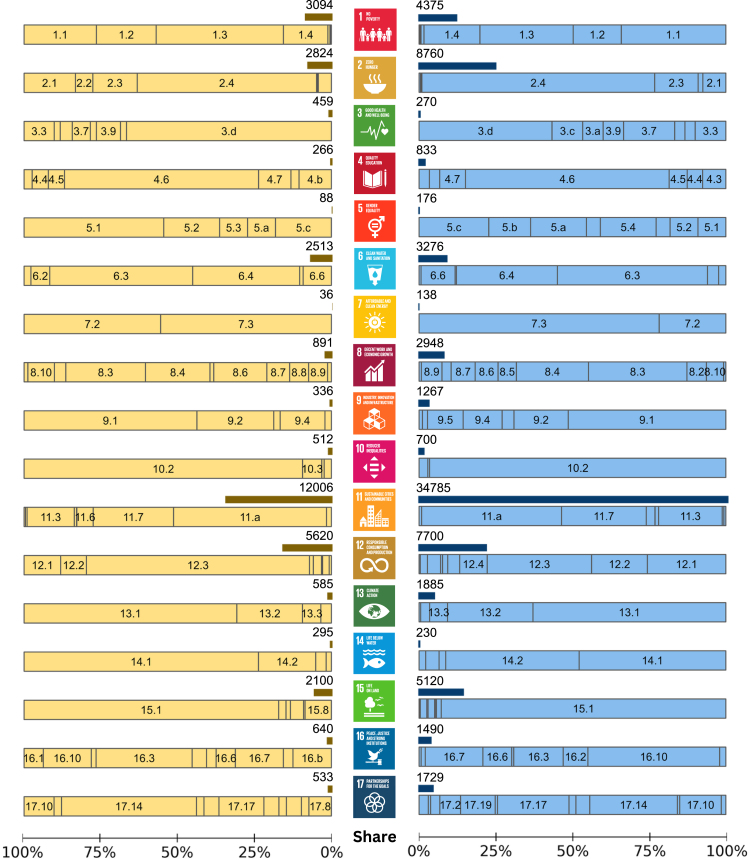
Negative (left) and positive (right) sentiments associated with the SDGs and targets mapped in the urban agriculture literature The darker color bar depicts the frequency of the mapped texts with positive or negative sentiments, considering more than two words associated with these sentiments (see experimental procedures for details). The light color bar represents the share of positive or negative sentiments among the targets within an SDG. It consists of the targets, e.g., 1.1, 1.2, and 1.3, with a share higher than 5%. Table S3 provides the full names of SDGs and targets.

Regarding sentiments, urban agriculture is more positively than negatively linked to SDGs (Figure 2). The mapped texts with positive sentiments are around double the negative ones, considering more than two words associated with these sentiments (see experimental procedures for details). All 17 goals are positively and negatively linked to urban agriculture, with more positive than negative ones, except for SDG 3 (*good health and well-being*). Studies highlight risks associated with food produced in urban areas, resulting in more negative sentiments for SDG 3. Urban agriculture has positive sentiments with 142 targets and negative sentiments with 136 targets. By analyzing the mapped text manually, we narrow these positive and negative associations to 81 and 51 targets. It is because words with positive or negative sentiments do not necessarily mean there are such connections. Thus, we assess, synthesize, and interpret the resulting associations to understand urban agriculture’s impacts on SDGs, extracting the reasons behind these sentiments (Tables S2 and S3). Table S3 elaborates on the reasons behind urban agriculture’s positive and negative impacts on SDGs at a target level. They include leveraging opportunities provided by the positive effects and hurdles to be resolved due to the negative ones of urban agriculture on sustainable development.

### Leveraging opportunities for SDGs

Urban agriculture mainly has positive impacts on many SDGs: 1, 2, and 4 (*quality education*); 5 (*gender equality*); 8 (*decent work and economic growth*); 9 (*industry, innovation, and infrastructure*); 10 (*reduced inequalities*); 11, 12, 13, 15, and 16 (*peace, justice, and strong institutions*); and 17 (*partnerships for the goals*). They represent the three sustainability pillars—society, economy, and environment—highlighting urban agriculture’s opportunities for broader sustainable development beyond enhancing food security. Table S3 details the reasons and mechanisms behind the positive associations of urban agriculture with SDGs at a target level. Here, we distill six factors behind these opportunities based on the most frequently compiled reasons for the positive impacts with some examples (Tables 1, S2, and S3).

**Table 1:** Leveraging opportunities and hurdles associated with urban agriculture, contributing to the achievement of various SDGs

Leveraging opportunities	SDG targets	Hurdles	SDG targets
Safe, nutritious, and fresh food	1.1, 1.4, 2.1, 2.2, 2.c, 8.2, 11.2, 12.3, 12.4, and 12.7	exclusive to the privileged groups	1.2, 1.5, 1.a, 1.b, 2.2, 4.1, 5.4, 5.a, 10.2, 13.3, 16.5, and 16.b
Knowledge and literacy	4.1, 4.3, 4.6, 12.6, 12.8, 13.3, and 15.7	contamination, pollution, and health hazards	1.3, 2.4, 3.3, 3.6, 3.9, 6.2, 8.4, 11.1, 11.2, 11.b, 12.4, 12.5, 14.2, 15.1, and 15.4
Ecosystem services, biodiversity, and environmental benefits	2.4, 2.5, 3.9, 6.1, 7.2, 8.4, 9.1, 11.1, 11.2, 11.3, 11.4, 11.5, 11.6, 11.7, 11.b, 12.1, 12.2, 13.1, 13.2, 13.b, 14.1, 14.2, 14.3, 14.4, 15.1, 15.2, 15.3, 15.4, 15.5, 15.6, 15.8, and 17.8	water security	6.1, 6.3, 6.4, 11.6, 13.1, 14.1, and 14.3
Social inclusion	1.b, 5.1, 5.4, 5.5, and 10.2	high energy and input usage	2.1, 2.3, 7.2, 7.3, 11.3, and 12.2
Employment and income	1.2, 1.3, 1.5, 1.a, 2.3, 4.4, 5.a, 6.2, 8.1, 8.3, 8.5, 8.6, 8.9, 8.10, 9.2, 9.3, 10.1, 12.b, and 17.1	others	1.1, 1.4, 2.5, 2.c, 8.1, 12.1, 15.2, 15.3, 15.5, 15.7, and 15.8
Psychological well-being	3.4 and 16.1		
Others	6.4, 11.a, 12.5, and 17.7		

We distill these opportunities and hurdles based on the most frequently compiled reasons behind the linkages between urban agriculture and SDGs in Table S3. “Others” consists of opportunities and hurdles linked with a few targets. They are partnerships, self-consumption, land tenure issues, illegal trade, recycling and reuse, urban renewal, water use efficiency, and ecosystem disservices. See Table S2 for the short names of the targets.

#### Safe, nutritious, and fresh food

Urban agriculture can produce and provide safe, nutritious, and fresh food within walking distance, positively impacting several SDGs, including ten targets (Table 1). By providing opportunities to produce their food, urban agriculture contributes to reducing food inequalities and malnutrition among underprivileged groups (targets 2.1, “universal access to safe and nutritious food,” and 2.2, “end all forms of malnutrition”). Examples of this contribution include an increased intake of healthy foods by black residents in Washington DC (USA),^25^ reduced malnutrition of children from underrepresented groups in Uganda, Bangladesh, and the Philippines,^20^ and provided essential micronutrients, e.g., Fe, Zn, Mo, I, and Ca, in Ghana.^26^ Urban farmers generate income by producing healthy and nutritious foods, reducing poverty (target 1.1, “eradicate extreme poverty”). For example, the Food and Agriculture Organization recognizes urban agriculture for sustainable poverty alleviation and food security, pointing to its role in improving the livelihoods of urban residents.^27^ Further, allotment gardens are an additional source of urban food. They can serve as a means to alleviate poverty by supplementing household food supply, reducing food costs, and enhancing food self-sufficiency.^28^ Increased physical activities of hobby urban gardeners improve their health, lowering healthcare costs associated with an unhealthy lifestyle.^28^ Producing and consuming local food decreases food miles and associated food wastage (target 12.3, “halve global per capita food waste”). For example, rooftop greenhouse cultivation in Barcelona (Spain) shows reduced food miles, resulting in lower packaging costs and less food waste.^2^^,^^29^^,^^30^^,^^31^ By composting, urban agriculture can use wasted food and agricultural residues to improve soil productivity and increase crop yield,^32^^,^^33^ contributing to target 12.4, “responsible management of chemicals and waste.”

#### Knowledge and literacy

Urban agriculture can improve urban populations’ knowledge and literacy (SDG 4) on food and nutrition, science and mathematics, environment and climate change (SDG 13), and responsible consumption and production (SDG 12). Table 1 provides the SDG targets associated with this leveraging opportunity. One of the main goals of urban agriculture initiatives is to create environmental awareness and education.^34^ For example, a study in Baltimore (USA) highlights the role of urban agriculture in providing city residents with opportunities to learn the provenance of food and to grow their food.^35^ Combining nutrition lessons with school gardening improves students’ knowledge of, preferences for, and consumption of fruit and vegetables in Pretoria (South Africa).^36^ Participation in school gardening increases students’ scores on science achievement tests, according to studies in the USA.^37^ Urban agriculture initiatives in Singapore use social media platforms to raise public awareness of sustainable food practices (targets 12.6, “encourage companies to adopt sustainable practices and sustainability reporting,” and 12.8, “promote universal understanding of sustainable lifestyles”).^38^ A study in Hamburg (Germany) highlights citizens learning about the effects of climate change (target 13. 3, “build knowledge and capacity to meet climate change”) by participating in urban agriculture.^39^ It also helps to acquire relevant technical and vocational skills (target 4.3, “equal access to affordable technical, vocational, and higher education”), potentially leading to future employment and income. For example, Scrubby Hill in Tasmania and a community greening program in Sydney (Australia) allow local communities to develop horticultural and landscaping skills to ensure employment, education, and career opportunities.^40^ Further, urban agriculture activities like foraging can play a role in urban ecosystem planning and management. Proper management and regulation of these activities can discourage poaching and trafficking of protected species by raising awareness and promoting sustainable harvesting practices,^41^ contributing to target 15.7, “eliminate poaching and trafficking of protected species.”

#### Ecosystem services, biodiversity, and environmental benefits

Urban agriculture is a part of urban green space (SDG 11), providing habitat for biodiversity and various ecosystem services (SDG 15), including noise reduction and improved air quality, and positively contributing to 32 targets (Table 1). For example, urban agriculture can create ample open spaces around housing developments, contributing to better housing typologies and living conditions^42^ (target 11.1, “safe and affordable housing”). Community urban gardens can act as inclusive and accessible green areas in cities, promoting social interaction among locals and improving the quality of life^43^^,^^44^ (target 11.7, “provide access to safe and inclusive green and public spaces”). Further, using agroforestry techniques in urban agriculture contributes to increased terrestrial carbon stocks^45^ and improved soil-based carbon sequestration as a climate change response (target 13.2, “integrate climate change measures into policies and planning”). Urban agriculture initiatives that convert waste and unused land into green spaces foster synergies between ecosystem services and climate regulation.^46^ If sustainable practices are adopted, urban agriculture could reduce chemical and material use and waste generation and save other natural resources (target 12.2, “sustainable management and use of natural resources”).^47^^,^^48^ Utilizing soil enrichment and land restoration techniques, urban agriculture can also combat desertification (target 15.3, “end desertification and restore degraded land”). Urban agriculture can be a part of green system integration to extend the life of physical infrastructure (target 9.1, “develop sustainable, resilient, and inclusive infrastructures”).^49^ Further, urban gardens have been demonstrated to be instrumental in preserving urban forests, thereby contributing to biodiversity conservation^43^^,^^46^ (target 15.1, “conserve and restore terrestrial and freshwater ecosystems”). The emerging practices of using ecological urban planning, called eco-tech cities, with more people using information communication and technology, encourage responsible living and green infrastructure, including urban agriculture (target 17.8, “strengthen the science, technology, and innovation capacity for least developed countries”). Such city projects are distributed worldwide, e.g., Milton (Canada), Waitakere (New Zealand), Helsinki (Finland), and Arcosanti (USA), which could improve energy and land resource use efficiency and increase the awareness of urban challenges.^50^

#### Social inclusion

Urban agriculture can promote social, economic, and political inclusion (SDG 10) for all, positively contributing to five targets (Table 1).^51^^,^^52^^,^^53^^,^^54^ It helps community building,^55^ reduces social exclusion, offers opportunities for alternative urban lifestyles,^56^^,^^57^ increases social cohesion,^58^ and fosters active civic participation.^59^ For example, a study from Bologna (Italy) highlights urban agriculture as a new community meeting point, providing an opportunity to reactivate relationships, cooperation, and solidarity, resulting in social inclusion and community building,^60^ contributing to target 10.2, “promote universal social, economic, and political inclusion.” Further, prioritizing communities in urban growth strategies through urban agriculture can help overcome the cycle of poverty and social vulnerability. Civic investments, often in education and other social infrastructure, play a key role in such strategies,^61^ contributing to target 1.b “create pro-poor and gender-sensitive policy framework.” Urban agriculture also promotes gender empowerment (targets 5.1, “end discrimination against women and girls”; 5.4, “value unpaid care and promote shared domestic responsibilities”; and 5.5, “ensure full participation in leadership and decision-making”). It helps change women’s traditional roles and power within a family with increased participation in household decision-making.^62^ Urban agriculture can also raise awareness of violence and discrimination against women.^63^ It provides an alternative political space where women can share and voice various oppression they experience,^62^ generating awareness and leaders.

#### Employment and income

Urban agriculture would create new jobs and income, contributing to many SDGs, including 19 targets (Table 1). It provides lower-income families with an additional source of income and savings for emergencies or crises,^64^ reducing poverty (target 1.2, “reduce poverty by at least 50%”)^27^^,^^65^^,^^66^^,^^67^ and inequality (target 10. 1, “reduce income inequalities”). Stimulating businesses that sell locally produced fresh food may attract private investors for infrastructures for urban agriculture,^49^ helping local communities to generate income and create jobs^68^ (target 8.2, “promote policies to support job creation and growing enterprises”). However, a huge initial cost associated with large-scale agriculture systems and land competition for other uses may discourage investors.^49^^,^^69^ The added income and jobs would also enhance urban farmers’ access to banking, insurance, and other financial services for their farming and way of life (target 8.10, “universal access to banking, insurance, and financial services”). In patriarchal societies where women are often restricted from working outside the home, urban agriculture, including indoor farming and home gardening, would provide employment opportunities for women (target 5.a, “equal rights to economic resources, property ownership, and financial service”). These opportunities can foster financial independence among women and contribute to narrowing the gender-based wage and employment gap.^70^^,^^71^ Furthermore, urban agriculture can promote modern farming techniques and advanced technologies to maximize crop production in limited spaces (target 9.2, “promote inclusive and sustainable industrialization”),^72^ e.g., aquaponics, rooftop greenhouses, and vertical farming systems. At a national level, public awareness of the ecosystem services provided by urban gardening and green space could be a new source of domestic tax revenue (target 17.1, “mobilize resources to improve domestic revenue collection”). For example, a South Korean study highlights Seoul inhabitants’ increased willingness to pay to enhance biodiversity in urban green spaces.^73^

#### Psychological well-being

Urban agriculture also promotes psychological well-being by lowering stress levels and increasing social interaction, contributing to a few SDGs, including two targets (Table 1). For example, a study of adult cancer survivors participating in urban gardening highlights improved mental health (target 3.4, “reduce mortality from non-communicable diseases and promote mental health”) due to reduced daily stress levels and an increased sense of a deep spiritual connection to nature.^74^ They also mentioned that the garden offers a venue for meeting and exchanging ideas and feelings, as well as a way to improve social networking. Besides improving mental health, urban agriculture can lower crime and gun violence (target 16.1, “reduce violence everywhere”) by promoting interaction, community involvement, and public presence.^75^ Mainly, urban green space significantly improves residents’ sense of community safety. The green areas in the urban setting encourage social interaction, community engagement, and public presence, which has positive psychosocial effects and reduces crime and violence.^75^

### Hurdles to be resolved

Depending on practices, urban agriculture can also negatively impact sustainable development, mainly SDGs 3, 6, 7 (*affordable and clean energy*), 11, 12, 14 (*life below water*), and 16. These practices include excessive agricultural inputs (e.g., water, energy, fertilizers, and pesticides), soil and water contamination, and non-mindful consideration of social injustices and inequalities. They create hurdles to leverage opportunities urban agriculture provides for broader sustainable development. Table S3 details the reasons behind the negative impacts of urban agriculture on SDGs at a target level. Here, we distill four factors behind these hurdles based on the most frequently compiled reasons for the negative impacts with some examples (Tables 1, S2, and S3).

#### Exclusive to the privileged groups

Urban agriculture initiatives will negatively impact several SDGs, including 12 targets (Table 1), if they do not address social injustices and inequalities. Doing so will hinder the realization of Leave No One Behind, a transformative SDG pledge, and exacerbate social exclusion (SDG 10) and discrimination (SDG 16). For example, neglecting social inequalities while implementing urban agriculture can reinforce the cycle of poverty and exclusion.^76^ Without an adequate financial mechanism, the privileged group might have an advantage over others in securing funds for urban agriculture, in contrast to target 16.b “promote and enforce non-discriminatory laws and policies.” For instance, in New York City (USA), urban agriculture initiatives led by white people reported raising more money to sustain their operations than initiatives led by people of color.^77^ Promoting urban agriculture could also lead to gentrification, displacing poor inhabitants and redistributing public investment and community capital toward the wealthiest part of the urban population.^78^ This displacement is opposite to target 10.2, “promote universal social, economic, and political inclusion.” Further, the privileged group might also influence urban agriculture policies to benefit them, excluding others’ needs. For example, another study in New York City (USA) highlights communities expressing concern about the proposed changes to municipal-level urban agriculture policy as they disregard communities of color and low income.^79^ Similarly, school garden programs are more likely to be a part of schools in wealthy neighborhoods than those of lower socioeconomic backgrounds.^37^ It would leave children from low-income communities behind in acquiring knowledge and literacy (target 4.1, “free primary and secondary education”) from school gardening. Furthermore, urban agriculture might create additional unpaid work on the top of care and domestic work of women^63^ with negative impacts on target 5.4, “value unpaid care and promote shared domestic responsibilities.”

#### Contamination, pollution, and health hazards

Urban agriculture could also be linked to contamination and pollution, resulting in health hazards and negative impacts on various SDGs, including 15 targets (Table 1). The main reasons behind the contamination and pollution are agriculture in or near industrial areas, urban pollution, contaminated water for irrigation, and heavy pesticide use. Consuming foods grown in polluted soil can cause a severe risk to human health (target 3.9, “reduce illness and death from hazardous chemicals and pollution”). Arsenic (As), cadmium (Cd), chromium (Cr), mercury (Hg), lead (Pb), copper (Cu), zinc (Zn), and nickel (Ni) are the heavy metals most frequently found in polluted urban soil, which crops and plants could uptake. For example, studies highlight exceeding the permissible limit of Pb, As, and Cd concentrations advised by the World Health Organization (WHO) and the Food and Agriculture Organization of the United Nations (FAO) in urban vegetables in Bangladesh.^80^^,^^81^ Crops and vegetables also absorb particulate matter containing heavy metals and polycyclic aromatic hydrocarbons suspended in the polluted urban air. Consuming these contaminated foods can cause cancer and harm the liver, kidneys, brain, bones, and lungs.^82^ Besides heavy metals, food grown in urban areas is more likely to be contaminated with bacteria if wastewater is used for irrigation. For example, high levels of microbial contamination, including fecal coliforms, fecal streptococci, and *Clostridium perfringens*, were found in the leafy vegetables in urban gardening at Porto-Novo (Benin).^83^ Urban agriculture could also foster waterborne diseases, e.g., malaria, by providing habitat in the irrigation systems for vector insects to breed, challenging target 3.3, “fight communicable diseases.”^84^ Studies also highlight risks of road safety (target 11.2, “affordable and sustainable transport systems”) and potential traffic accidents (target 3.6, “reduce road injuries and deaths”) due to urban agriculture, mainly related to poor visibility^85^ and road verge gardens.^86^ Furthermore, composting for urban agriculture can lead to ammonia generation and acidic leachate. Also, urban farmers may improperly use chemical fertilizers and herbicides without awareness, negatively impacting target 2.4, “sustainable food production and resilient agricultural practices.”

#### Water security

Without adequate management, promoting urban agriculture would be a water security concern, negatively impacting several SDGs and seven targets (Table 1). Using groundwater and municipal water to irrigate urban agriculture could result in overexploitation of the aquifer,^70^ inefficient freshwater supplies^87^ (targets 6.1, “safe and affordable drinking water,” and 6.3, “improve water quality, wastewater treatment, and safe reuse”), and tensions related to water use. Excessive use of fertilizers and pesticides in urban agriculture would pollute water. Thus, disposing of wastewater from urban agriculture without treatment could degrade water quality, including groundwater,^88^ and pose a threat of agrichemical to microbiological contamination.^89^ Doing so can result in contaminated drinking water sources,^90^ pose health risks, cause harm to marine ecosystems (targets 14.1, “reduce marine pollution,” and 14.3, “reduce ocean acidification”),^91^ and pollute water bodies and ecosystems.^92^^,^^93^ Urban agriculture management, including compost production and specific inputs, e.g., wood and peat, can also cause the eutrophication of water bodies due to leaching.^91^ Urban agriculture on soils containing polycyclic aromatic hydrocarbons can harm human health and the environment, possibly contaminating marine ecosystems if they enter the sea through runoff or leaching.^94^ These potential negative impacts of urban agriculture are also against target 11.6, “reduce the environmental impact of cities.” Further, urban agriculture may also stress the city’s water sources and supply, hindering its ability to adapt and be resilient to natural hazards (target 13.1, “strengthen resilience and adaptive capacity to climate-related disasters”).^87^

#### High energy and input usage

Urban agriculture could negatively impact many SDGs, including six targets (Table 1), because of high energy and input uses. Conditioned urban agriculture demands energy to regulate and maintain optimal environmental conditions, including lighting, temperature, and humidity control.^95^ This energy demand could impact climate change more than conventional agriculture.^96^ It could also increase reliance on non-renewable energy sources^86^ (targets 7.2, “increase global percentage of renewable energy,” and 7.3, “double the improvement in energy efficiency”). For example, heated greenhouse-produced tomatoes in Boston (USA) have potentially higher impacts on climate change and non-renewable resource depletion than imported ones.^2^ Rooftop urban agriculture also competes with solar panels for valuable space.^97^ Unsustainable urban agriculture, as opposed to target 12.2, “sustainable management and use of natural resources,” has issues with excessive use of pesticides, fertilizers, and water, resulting in health risks and environmental problems.^98^ For high-tech urban farming, lacking these inputs would limit urban agriculture’s contributions to targets 2.1, “universal access to safe and nutritious food,” and 2.3, “double the productivity and incomes of small-scale food producers.”^99^

## Discussion

Our systematic text analysis highlights more positive than negative impacts of urban agriculture on sustainable development beyond food security. Leveraging these positive impacts could enhance urban agriculture’s contribution to sustainability. However, resolving hurdles generating urban agriculture’s negative impacts is crucial for maximizing the benefits from the positive ones. Thus, our study brings several novelties and insights into urban agriculture’s transformative opportunities to promote sustainable development, including the hurdles to be resolved.

First, our study synthesizes a holistic understanding of the impacts of urban agriculture on SDGs at the target level. Advancing the existing studies,^1^^,^^9^^,^^21^^,^^100^ we compile evidence from the literature on multiple benefits of urban agriculture across social, economic, and environmental dimensions and its limitations. Promoting urban agriculture could positively impact SDGs 1, 2, 4, 5, 8, 9, 10, 13, 15, and 17. However, the current urban agriculture practices could also negatively impact SDGs 3, 6, 7, and 14, with both positive and negative impacts on SDGs 11, 12, and 16. These findings underscore that the inherent contribution of urban agriculture to sustainability is contingent on specific practices. Nevertheless, urban agriculture presents numerous transformative opportunities to foster broader sustainable development.

Second, we extract the key factors leading to the positive impacts of urban agriculture and its transformative opportunities from the existing literature. Promoting sustainable urban agriculture would help leverage these opportunities for making progress across SDGs. Our systematic text analysis synthesizes these opportunities, fragmented across the literature. For example, urban agriculture can produce safe, nutritious, and fresh food within and nearby cities, potentially providing a larger share of urban vegetable demand.^101^^,^^102^^,^^103^ Doing so generates employment and creates additional income for urban inhabitants and farmers.^104^^,^^105^ Besides food, urban agriculture provides various ecosystem services^1^ and harbors biodiversity.^43^^,^^106^^,^^107^^,^^108^ It would generate knowledge and literacy on various aspects, including healthy diets, environmental issues, and sustainability.^34^^,^^36^^,^^109^ Urban agriculture promotes social inclusion and community building^110^ and enhances psychological well-being.^111^ Compiling these opportunities urban agriculture provides is crucial for a holistic understanding to leverage them for accelerating SDG progress.

Third, our evidence synthesis emphasizes that resolving hurdles behind urban agriculture’s negative impacts is crucial for leveraging transformative opportunities. For example, the health benefits of urban-produced fresh food can only be obtained by tackling the issues of soil and water contamination, urban pollution, and overuse of pesticides. Failure to address these issues may lead to adverse health consequences from consuming urban-produced food. The social benefits of urban agriculture risk being compromised if policies favor privileged groups and neglect considerations of gender, ethnicity, and other aspects of intersectionality.^112^ Ensuring the environmental benefits of urban agriculture necessitates sustainable farming practices. Otherwise, it may inadvertently harm the environment and ecosystems instead. These practices include efficient water use for irrigation, proper treatment of wastewater generated from urban agriculture, efficient energy and input uses, and combining conditioned urban agriculture with renewable energy.

Fourth, our text analysis approach systematically helps capture the mechanisms behind SDG interactions in future studies. We combine systematic literature screening, mining relevant text on SDGs, exploring sentiments on the extracted text, and in-depth manual text analysis for qualitatively understanding the mechanisms at the target level. In other words, our approach supplements computer-based analyses with in-depth manual analysis. Our computer-based programs save time in narrow down the texts associated with SDGs in the relevant documents. The in-depth manual analysis of these narrowed-down texts helps filter miss-classified sentiments and not-so-relevant texts, assessing, compiling, and synthesizing the positive and negative links between urban agriculture and SDGs with their underlying reasons and mechanisms. So far, limited qualitative studies have investigated SDG interactions by systematically covering a range of literature^13^^,^^113^ or at the target level.^9^ A robust understanding of the mechanisms of SDG interactions requires systematic studies, including evidence synthesis from the literature.^18^

In the meantime, our study also has a few limitations. Our analyzed literature could have been updated as the initial search was done on 15.02.2022. However, this literature covers an extensive body of literature on urban agriculture, and we also include recent literature to contextualize our study. There might still be authors’ bias while extracting the information from the literature on the impacts of urban agriculture on SDGs. We minimize the biases by using text mining and sentiment analysis before analyzing them manually. Also, our sentiment analysis is limited to the word list used to identify positive or negative associations. However, words with positive or negative sentiments do not necessarily mean there are such connections. We address this limitation by conducting an in-depth manual analysis of the mapped text. Further, we apply the four-eyes principle to ensure the robustness of the manual analysis based on reviews by coauthors. We do not focus on the impacts of SDGs on urban agriculture, which could be a question for future research. The achievement of some SDGs could promote sustainable urban agriculture.

In summary, urban agriculture can have broader positive social, economic, and environmental impacts by implementing sustainable practices. These practices are essential to resolve its current negative impacts, which include sustainable farming, addressing urban pollution, and ensuring social justice and fairness. Furthermore, the issues of land ownership, accessibility, and availability could hinder the successful implementation and sustainability of urban agriculture initiatives. These issues are mainly related to rapid urbanization, unfavorable policies, and a high value of urban land.^114^^,^^115^ A high installation cost is another challenge to promoting urban agriculture, e.g., in the case of conditioned one. Therefore, leveraging urban agriculture’s social, economic, and environmental benefits requires sustainable practices and tackling these challenges. Specific practices to promote urban agriculture can be context-specific and vary across urban areas, which is a topic for further investigation and innovation.^116^

## Experimental procedures

### Data

Our analysis makes use of two datasets. The first is the data on relevant literature on urban agriculture. Since identifying relevant literature from databases is daunting, we base our analysis on recently searched and screened literature on urban agriculture by Pradhan and colleagues.^9^ They identified 76,000 records of urban agriculture literature by systematically exploring the two well-established databases—Web of Science and Scopus. They applied a broad keyword search strategy with words “∗urban∗ or city or cities” and “agricultur∗ or garden∗ or farm∗ or food” and “form∗ or type∗ or typolog∗ or class∗ or catagor∗ or kind∗” in the title, abstract, or keywords. They applied a machine learning-supported approach to screen the records by reading their titles, abstracts, and keywords. This approach includes screening a subset of records to select relevant documents manually, using this subset to train the supervised machine learning algorithms for prioritizing documents likely to be relevant from the remaining records,^117^ re-training the algorithms while screening documents, and using stopping the screening when it is unlikely to remain to identify more than 5% of relevant documents.^118^ In doing so, they collected and screened the complete text, i.e., portable document format (PDF), of the relevant documents, resulting in 1,455 relevant full texts. We use these PDFs for our analysis.

We develop search query data for mapping SDGs in the relevant documents at the target level (Table S1). For this, we adopt the search queries provided by the Aurora Universities Network.^23^ The provided search queries comprise sub-queries for each SDG target, constructed with keywords, boolean, and proximity operators and tailored to the Scopus Query Language. We converted these queries to Corpus Query Language. Doing so enables us to apply these queries using the corpustools package in the R programming language. We also adjust the proximity operator to locate one word within an increased distance of another after testing different word distances. Since we analyze the text in PDFs containing up to three columns, it is essential to increase the word distance. A short word distance will not map the relevant text to SDGs if it is in the following line, separated by columns. By contrast, a long word distance may also map SDGs to irrelevant text. Table S1 contains the developed SDG search queries used for our analysis.

### Text analysis

Our text analysis consists of computer-based programs and in-depth manual analysis. It starts by mapping each SDG target in the relevant documents using the corpustools package in R.^24^ Our mapping focuses on understanding how well different SDGs and targets are represented across the literature. This mapping is followed by a sentiment analysis to determine urban agriculture’s positive or negative association with SDGs. We supplement these computer-based analyses with in-depth manual analysis to assess and synthesize the mapped text, referring to the relevant documents. Our computer-based programs help to narrow down the texts associated with SDGs in the relevant documents without the need to read the full articles, saving time and resources. The in-depth manual analysis addresses the limitations of computer-based programs in text analysis. Computer-based programs might miss-classify sentiments and extract not-so-relevant texts in terms of the objective of a search query. Thus, our text analysis approach consists of three steps.

First, we map SDGs in the relevant documents at the target level using the corpustools package in the R. The package provides text analysis in a tokenized text format, breaking up a given text into units called tokens.^24^ Since our search queries are based on words, we tokenize individual words. We map each SDG target to each relevant document based on the search queries by applying “search_features” and “get_kwic” commands of the package. Using these commands, we extract 50 tokens for each query search result to have enough text for further manual analysis. These tokens are the text in the relevant document mapped to an SDG target. Compiling these extracted texts, we obtain a database of the mapped text for each SDG target from the relevant documents. In addition to these tokens, the database consists of the document’s identification number, the mapped text’s page number, and the search query. We count the frequency of the mapped text per SDG and target to capture how well different SDGs and targets are represented across the literature.

Second, we conduct a sentiment analysis to determine if the mapped text highlights urban agriculture’s positive or negative association with SDGs using the tidytext package in R. It provides a list of words associated with different sentiments. Since the words “sustainable,” “sustainability,” “poor,” and “poverty” are used to describe some SDG targets and are not necessarily associated with positive or negative sentiments, we exclude them from the list. Afterward, we update our database using the command “get_sentiments” from the package to obtain positive or negative sentiments associated with the mapped texts and the words behind these sentiments. We consider positive and negative associations when the words behind these sentiments are more than two words. We further use the text mapping and sentiment analysis results to manually assess and compile the reasons behind urban agriculture’s positive or negative impacts on SDGs.

Third, we conduct an in-depth manual analysis of the mapped text and its sentiments to understand the reasons behind urban agriculture’s positive or negative impacts on SDGs. Since the map texts are fragmented, we refer to the relevant document and assess and synthesize the sentences behind these map texts. We distribute the SDGs among the coauthors for the manual analysis, with a coauthor responsible for at least one SDG. Based on our in-depth manual analysis, we extract the reasons behind urban agriculture’s positive or negative impacts and compile them for each SDG target. We exclude the mapped texts unrelated to SDGs and urban agriculture during our manual analysis. We apply the four-eyes principle to ensure the robustness of the manual analysis. A coauthor, not responsible for that SDG, reviews the extracted reasons behind the impacts. Lastly, we extract leveraging opportunities provided by the positive linkages and hurdles to be resolved due to the negative association of urban agriculture with sustainable development based on the most frequently compiled reasons at the target level.

## Resource availability

### Lead contact

The lead contact is Prajal Pradhan: p.pradhan@rug.nl.

### Materials availability

No specific material was used for this study.

### Data and code availability

Search query data for mapping SDGs is provided as a supplemental item (Table S1). Other data reported in this paper will be shared by the lead contact upon request. This paper does not report the original code. Any additional information required to reanalyze the data reported in this paper is available from the lead contact upon request.

## Acknowledgments

P.P. thanks the Food System Economics Commission, funded by the IKEA Foundation (grant agreement no. G-2009-01682) and acknowledges funding by the European Research Council (ERC) for the BeyondSDG project (Project number 101077492) and the German Academic Exchange Service (DAAD) for the ForHimSDG project (Projekt-ID 57611837) with financial support from the Federal Ministry for Economic Cooperation and Development of Germany (BMZ). Y.H. thanks the China Scholarship Council (202206275002) and the National Natural Science Foundation of China (grant 52100214). Many thanks go to Jan Minx for providing exposure to various techniques for evidence synthesis. We thank Manoj Gyawali, Sanderine Nonhebel, Diego Rybski, and Anne Warchold for their valuable feedback on our study.

## Author contributions

Conceived and designed the experiments: P.P.; performed the experiments: P.P.; analyzed the data: P.P., D.R.S., K.D., Y.H., P.G., S.P., S.K., and B.K.; contributed materials/analysis tools: P.P., D.R.S., K.D., Y.H., P.G., S.P., S.K., B.K., S.B., M.G., and A.J.; wrote the paper: P.P., D.R.S., K.D., and Y.H.

## Declaration of interests

P.P. is an advisory board member of *Cell Reports Sustainability*.

## Supplemental information

Table S1. Corpus query strategy for mapping SDGs at the target level adopted from the Aurora Universities Network A target consists of more than one query strategy because of its multiple indicators and aspects.

Table S2. Leveraging opportunities and hurdles associated with urban agriculture, contributing to the achievement of various SDGs These opportunities and hurdles are distilled from the most frequently compiled reasons behind the linkages between urban agriculture and SDGs in Table S2. “Others” consists of opportunities and hurdles linked with a few targets. They are partnerships, governance issues, recycling and reuse, urban renewal, water use efficiency, and ecosystem disservices. The short names of the targets are provided in the brackets.

Table S3. Mechanisms behind impacts of urban agriculture on SDGs at the target level These mechanisms are grouped into positive and negative ones and systematically extracted from the literature using a text analysis approach. In doing so, we exclude the mapped texts unrelated to SDGs and urban agriculture during our manual analysis, resulting in NA (not available) links for some SDG targets. We consider UA as farming in urban and peri-urban areas. Each SDG is presented in a separate tab. The last tab consists of references for all the SDGs.
